# The role of extracellular vesicles in rheumatoid arthritis: a systematic review

**DOI:** 10.1007/s10067-021-05614-w

**Published:** 2021-02-05

**Authors:** Tommaso Schioppo, Tania Ubiali, Francesca Ingegnoli, Valentina Bollati, Roberto Caporali

**Affiliations:** 1 Division of Clinical Rheumatology, ASST Pini-CTO, Piazza Cardinal Ferrari 1, 20122 Milan, Italy; 2 Department of Clinical Sciences and Community Health, Research Center for Adult and Pediatric Rheumatic Diseases, Research Center for Environmental Health, Università degli Studi di Milano, Milan, Italy; 3 EPIGET LAB, Università degli Studi di Milano, Milan, Italy

**Keywords:** Exosomes, Extracellular vesicles, Microvesicles, Rheumatoid arthritis

## Abstract

Rheumatoid arthritis (RA) is a chronic inflammatory disease that carries high social and economic costs and can lead to permanent disability. RA pathogenesis has not been completely elucidated yet. Extracellular vesicles (EVs) are membrane-contained vesicles released by cells playing a role in cell-to-cell communication and they could be involved in different diseases. Evidence on the involvement of EVs in RA is currently inconclusive. Therefore, a systematic review on the role of EVs in RA was performed in order to explore this relationship. This review followed the Preferred Reporting Items for Systematic Reviews and Meta-Analyses (PRISMA) guidelines. The research was conducted on PubMed, Scopus, and Embase up to March 5, 2020: 41 studies were analyzed out of 674 screened. The total plasmatic and synovial fluid (SF) EV number seems increased in RA as compared with healthy controls. Both RA plasma and SF contained EVs subpopulations of heterogenous origin, especially derived from platelets and immune system cells. No univocal evidence emerged on miRNA expression and EV content profile within RA patients. EVs showed to enhance pro-inflammatory pathways, such as cytokines and chemokine release and TNF blockade seemed to revert this effect. Our work highlights the requirement to standardize study methodologies in order to make results comparable and draw conclusions that remain, at present, unclear.

**Supplementary Information:**

The online version contains supplementary material available at 10.1007/s10067-021-05614-w.

## Introduction

Rheumatoid arthritis (RA) is a chronic inflammatory autoimmune disease with a considerably high social and economic impact. RA can result in loss of function, permanent disability, and severe systemic complications, such as cardiovascular events. RA affects about 0.5–1% of the general population worldwide and it involves any age group, with a predominance for the third, fourth and fifth decades [1]. RA is still considered an incurable disorder, even if disease remission can be obtained with tight control and treat-to-target strategies, as suggested by current recommendations [2]. Disease flares occur in more than half of the patients and they substantially contribute to radiographic damage, poorer quality of life, disability, healthcare use, and costs [3, 4]. Presently, there are no predictors of therapy response and no indications about personalized treatment.

Despite recent advances, RA pathogenesis has not been completely elucidated yet. The genetic background plays a relevant role in RA susceptibility, but its contribution to the pathogenesis is partial [5]. Besides this, environmental factors, such as cigarette smoke and air pollution, have been identified as potential triggers for RA [6–8].

Extracellular vesicles (EVs) are membrane-contained vesicles released by cells in all biological fluids and they have been described as impaired in many pathological conditions, such as RA. EVs can transmit molecular effectors to other cells, thereby affecting the recipient cell function. EVs can be classified into three main groups: microvesicles, formed by external budding and fission of the plasma membrane; exosomes, produced within the cell and set loose after fusion of vesicular bodies with the plasma membrane; apoptotic bodies, released like blebs of apoptotic cells [9]. Studies on EVs made use of different terminology to describe EVs and this could pose difficulties for a direct comparison between them. For this reason, in our review, according to the last version of the position statement of the International Society for Extracellular Vesicles [10], we use the term EVs as a generic term to include the whole group of EVs.

Plasmatic EVs have been proposed as potential biomarkers. Furthermore, it has been suggested that EVs, after internalization into target cells through surface-expressed ligands, may transfer miRNAs, enabling inter-cellular and inter-organ communication [11]. To our knowledge, although several reviews have been published so far, none included a full systematic revision of the literature about EVs in RA [12–15].

It was against this background that we sought to explore the role of EVs in RA to present a complete and comprehensive recap about the state of the art on this topic.

## Materials and methods

### Protocol and registration

This review was conducted according to the Preferred Reporting Items for Systematic Reviews and Meta-Analyses (PRISMA) guidelines for reporting systematic reviews and meta-analyses [16, 17]. Our protocol was registered on PROSPERO (CRD42020181164) [18].

### Literature search

A comprehensive systematic literature search was undertaken using PubMed, Embase, and Scopus. The search strategy was planned to capture all the studies focusing on EVs in patients with RA with no restrictions for sex or therapies. The search terms were adapted according to bibliographic databases in combination with database-specific filters, where these were available. The full search strategy is detailed in supplementary materials (table S1).

### Eligibility criteria

Inclusion criteria: studies including adult patients with RA and investigating EVs, regardless of the technique applied. Clinical trials, observational studies (cross-sectional, prospective, and retrospective), case series (if subjects were ≥ 5), studies with at least an abstract in English, and studies published from database inception to March 2020 were included.

Exclusion criteria: studies on patients under 18 years of age, review articles, animal or cell models, case reports or case series with ≤ 5 subjects, and editorials were excluded.

### Data extraction and synthesis

The research was performed on 5 March 2020. The citations were imported into the reference management software package Endnote X8. Duplicated references were automatically eliminated both by the Endnote software and manually by two reviewers (T.U. and T.S.).

During the first screening, the two reviewers independently screened titles/abstracts from the list of records retrieved, and full papers were sought when abstracts were felt to be relevant. Moreover, reference lists of the reviewed articles were examined for relevant studies. In cases of disagreement, a decision was made by consensus.

The two investigators, then, independently analyzed the full-text papers and extracted the relevant data from the included studies in standardized data extraction forms. The two authors then crosschecked the extracted data to rule out any discrepancies. Unresolved disagreements between two reviewers were resolved by consensus. The following data were evaluated: first author's surname, publication year, country, study design, setting, diagnostic criteria, outcomes measured, patient enrolment strategies, methods for EVs analysis, participant characteristics (age, gender, therapies), and results.

## Results

### Literature review

**Fig. 1:**
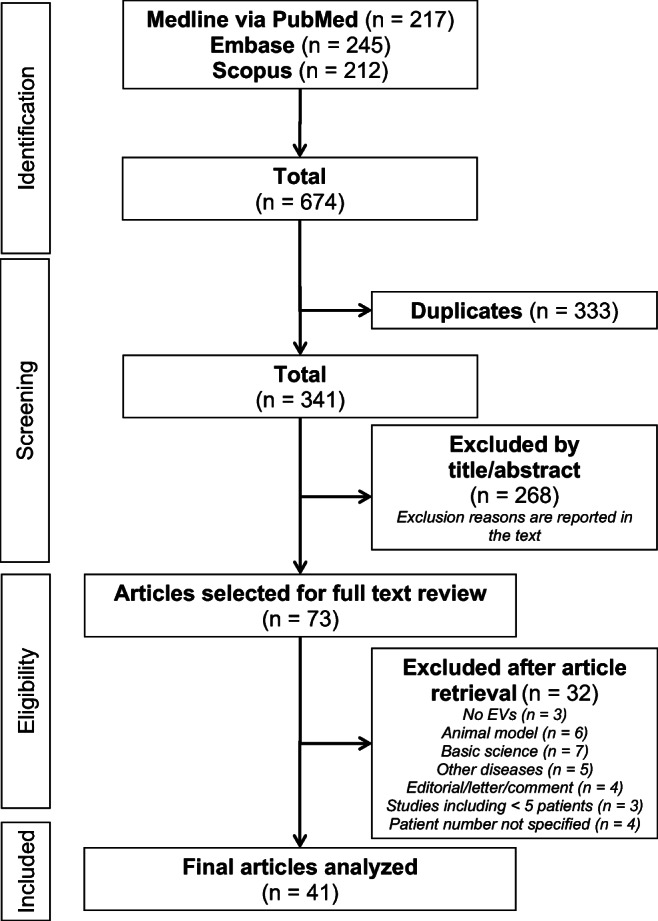
PRISMA Flow diagram illustrating literature research and selection process

In total, 674 references were retrieved in the initial search strategy in Medline via PubMed, Embase, and Scopus. 333 references were excluded as duplicates. 268 references were excluded after title/abstract screening. Exclusion reasons were: studies not considering EVs (*n* = 61), basic science studies (*n* = 40), studies not considering RA (*n* = 102), type of articles (editorial/letter/comment/book; *n* = 55), case reports (*n* = 8), duplicated (*n* = 2). 73 articles were retrieved for full paper review of which 41 references fulfilled the inclusion criteria. Thirty-two manuscripts were excluded (exclusion reasons are reported in Fig. 1). The review flow process is outlined in Fig. 1. All the findings of the studies included in the present systematic review are reported in Table 1 and supplementary materials (table S2).

**Table 1:** Characteristics of the included studies

References	Diagnosis and patient number	RA therapies	Findings
Rodríguez-Carrio J. (2015) [19]	114 RA** 33 HC 72 CV risk	None or NSAIDs: 10.5%; GC: 53.5%; MTX: 70.1%; TNFi: 39.4%; TCZ: 10.5%	Total MPs: RA 4.21 vs. HC 2.1 (*p* < 0.0001) vs. CV risk 3.14 × 10^6^/ml (*p* = 0.001) CD146+, CD66+, CD3CD31+ MPs were increased in RA vs. HC (*p* = 0.029, *p* = 0.001, *p* < 0.001) RA total MPs were associated with traditional CV risk factors and with the number of CV risk factors CD146+ was associated with disease duration (*p* = 0.005); CD66b+ with DAS28 (*p* = 0.032), ESR (*p* = 0.022), age at diagnosis (*p* = 0.021); CD3+CD31+ with DAS28 (*p* = 0.007), TJC (*p* = 0.026), SJC (*p* = 0.003); CD14+ with RF (*p* = 0.041) Patients in TCZ: lower CD3+CD31+ and CD66b+ (*p* = 0.005; *p* = 0.011) Patients in MTX: lower CD3+DC31+ (*p* = 0.033) TNFα correlated with CD3+CD31+ (*p* = 0.097) if no traditional CV risk factors (*p* < 0.0001). At multivariate regression model including CV risk factors, TNFα was associated with CD3+CD31+ (*p* = 0.012) MPs from RA had a dose-dependant anti-angiogenic effect (CD14+ and CD41+ MPs) and endothelial activation (CD62E, CD144, VEGFR)
Arntz O.J. (2018) [20]	41 RA** 24 HC	DMARDs: 75%; GC: 53.7%; bDMARDs: 19.5%	No difference in size, protein content concentration, concentration plasmatic EVs in RA vs. HC No difference in size, content, concentration plasmatic EVs in RF+ vs. RF- (no difference between disease parameters apart from ESR higher in RF+, *p* < 0.05) In RF+: 13/28 were found to have IgM-RF in EVs. Patients with IgM-RF on EVs had higher VAS, CRP, DAS28 and ESR (*p* < 0.001, *p* < 0.01, *p* < 0.05, *p* < 0.01), no difference for TJC and SJC
Skriner K. (2006) [21]	5 RA* 5 ReA 5 OA	–	Similar amount of exosomes in all patients Citrullinated and non-citrullinated proteins present in all samples Fibronectin/IgG immune complex only in RA exosomes
Atehortùa L. (2019) [22]	9 RA** 9 SLE 6 HC	–	Endothelial cells internalized MPs and MPs-IC Macrovascular HUVEC + MPs/MPs-IC ➔ increase ICAM-1, ICAM-2, IL-6 and IL-8 (dose dependant for ICAM-1, IL-6 and IL-8) Microvascular HMVEC-L + MPs-IC ➔ increase in ICAM-1 Microvascular HMVEC-L + MPs ➔ increase in CCL2 Microvascular HMVEC-L + MPs/MPs-IC ➔ increase in CCL5 No effect of MPs/MPs-IC on HMVEC-D HUVEC + MPs/MPs-IC ➔ increase of adhesion of classical monocytes HUVEC + MPs ➔ increase of adhesion of non-classical monocytes (not for MPs-IC) HMVEC-L + MPs/MPs-IC ➔ decrease of adhesion of classical monocytes MPs and more MPs-IC altered endothelial monolayers (micro- and macrovasculature) with increased permeability of macrovascular endothelial cells
Barbati C. (2018) [23]	20 (+10) RA** 20 HC	ETN 50 mg SC weekly + csDMARDS: 20; csDMARDs: 10	Total MPs and endothelial MPs higher at baseline in RA vs. HC (*p* < 0.0001) (no difference for platelet and leucocyte MPs) After ETN: total MPs and endothelial MPs decreased from baseline (*p* < 0.0001 and *p* = 0.03) At baseline TNFα was more expressed on MPs in RA than HC (*p* = 0.0009) with a decrease after 4 months of ETN (*p* = 0.0002) (no change for patients treated only with csDMARDs) *In vitro* experiment: MPs-TNFα decreased dose-dependently after incubation with ETN Significant correlation between MPs-TNFα and DAS28, TJC, SJC, CDAI, and HAQ RA-MPs increased dose-dependently apoptosis and autophagy with respect to untreated cell (*p* = 0.005 and *p* = 0.02)
Birò E. (2007) [24]	10 RA* (8 for SF, 9 for plasma) 10 HC	–	Complement activator products (C4b/c and C3b/c) in RA SF were higher than RA plasma and HC (*p* < 0.05, *p* < 0.01, *p* < 0.01, *p* < 0.01); no difference between RA plasma and HC SAP and IgG in RA SF were lower than RA plasma and HC (*p* < 0.001, *p* < 0.001, *p* < 0.01, *p* < 0.01); no difference between RA plasma and HC CRP was higher in RA plasma than HC (*p* < 0.001); no difference between SF and plasma IgM showed no difference between all groups MPs were higher in RA SF than HC plasma (*p* < 0.05); no difference between RA plasma and HC or RA SF and RA plasma MPs with C1q, C4, and C3 were higher in RA SF than RA plasma and HC plasma (*p* < 0.01; *p* < 0.05); no difference between RA plasma and HC plasma MPs with CRP and SAP showed no difference between groups MPs with IgM and IgG were higher in RA SF than RA and HC plasma (*p* < 0.05, *p* < 0.01, *p* < 0.01, *p* < 0.01)
Boilard E. (2010) [25]	20 RA 20 OA 6 JIA 19 PsA 14 Gout	–	Platelet MPs are abundant in inflammatory SF (no statistical significance provided)
Burbano C. (2018) [26]	60 RA** anti-CCP^−^RF^−^ = 6 anti-CCP^+^RF^+/−^ = 26 anti-CCP^hi^RF^hi^ = 28 40 HC	No bDMARDs	Plasma EV count: statistically different between HC anti-CCP^+^RF^+/−^ (no difference for seronegative and anti-CCP^hi^RF^hi^) EV size distribution: anti-CCP^+^RF^+/−^ and anti-CCP^−^RF^−^ had decreased proportions of 0.1–1 μm and elevated proportions of 1–3 μm and 3–6 μm EV as compared with HC Cellular source: seropositives had more CD41a+ EVs and seronegatives had elevated CD105+ EVs EV components: EV-ICs and EV-CPs were significantly elevated in seropositives as compared with HC. Anti-CCP^+^RF^+/−^ had higher C1q EVs and HMGB1 than other groups. EVs of seronegative patients were similar to HC. EV-ICs, EV-CPs, EV-C1q, and EV-HMGB1 derived from platelets and leukocytes (more from platelets in seropositives). EVs from seropostives had higher frequencies and wider distribution of EV-IgM+ and EV-IgG+ (on 5 patients for group) EVs positive for IgG, IgM, CD41a, and citrulline were associated with systemic inflammation in seropositive patients EVs from seropositive patients could activate mononuclear phagocytes and induce pro-inflammatory cytokines (TNFα, IL-6, and IL-1beta)
Burbano C. (2019) [27]	34 RA** (according to DAS28: 28 in remission and 18 in moderate activity) 34 SLE 14 HC	No bDMARDs	MP-ICs promote MDM differentiation to a pro-inflammatory profile (M1-like) more evident in SLE and RA than HC. MDM differentiated with MP-ICs from RA patients were resistant to repolarization to M2-like after IL4- treatment. MDM differentiated with MP-ICs from RA patients enhanced T cell proliferation, B cell activation markers, and B cell death prevention (not found for IFNγ, TNFα, and other B cells parameters)
Berckmans R.J. (2002) [28]	10 RA* 10 non-RA arthritis 20 HC	RA: number of DMARDs 2.6 (0–5); non-RA: number of DMARDs 1.5 (1–3)	MPs from RA plasma, but not from SF, strongly bound annexin V Similar results for non-RA MPs from platelets were absent in SF but were the most abundant in plasma (*p* < 0.0001). The opposite was for granulocytes and monocytes (*p* = 0.0001, *p* < 0.0001). In SF, MPs from CD4+, CD8+, B cells and erythrocytes were low (*p* = 0.0001, *p* = 0.0002, 0=0.041, *p* = 0.0001). No difference for RA and non-RA. Thrombin-generating capacity (factor VIIa) for SF MPs was higher than patients’ and HC plasma TF was absent on SF MPs despite they initiated TF-mediated thrombin generation Patients’ plasma vs HC: prothrombin fragment F_1+2_ and thrombin-antithrombin were increased (*p* < 0.0001, *p* = 0.0003). No difference for RA and non-RA SF (*p* = 0.16 and 0.26). Higher levels in SF as compared with patients’ plasma (*p* < 0.0001, *p* < 0.0001). Higher levels in RA plasma than non-RA plasma (*p* = 0.004, *p* = 0.0003)
Michael B.N.R. (2019) [29]	40 RA** 33 seropositive 7 seronegative 35 young-onset 15 extra-articular manifestations 30 OA 33 HC	No therapy, GC included	SF from RA vs. OA: more annexin V, leucocyte-derived, monocyte-derived, granulocyte-derived MPs CD4+ and CD8+ MPs (*p* < 0.001, *p* < 0.001, *p* < 0.001, *p* < 0.001), B cell–derived not detectable in both Granulocyte-derived MPs were more elevated in established RA SF than early RA (*p* = 0.03), annexin V MPs and platelet-derived MPs were increased in RA SF with extra-articular manifestations (*p* = 0.02, *p* < 0.011), ACPA positive RA patients had more SF granulocyte MPs than ACPA negative (*p* = 0.02). There was a weak correlation between ACPA titer, CD4 MPs and granulocyte-derived MPs Plasma annexin V and leucocyte-derived MPs were different in RA, OA and HC (*p* < 0.001, *p* < 0.001). RA plasma had more annexin V, leucocyte-derived, platelet-derived MPs, and CD61 as compared with OA and HC (*p* < 0.01, *p* < 0.001, *p* < 0.01, *p* < 0.001, *p* < 0.001, *p* = 0.02, *p* < 0.001). OA plasma had more annexin V, CD61 MPs vs. HC (*p* = 0.002, *p* = 0.01). Leucocyte-derived MP sub-populations (CD20, CD4, CD8, CD14, CD66b) were not detectable in plasma No difference emerged for plasma MP profile among clinical and serological RA phenotypes
Chen Z. (2018) [30]	11 RA** 11 OA	–	MMP14 and VEGF expression were higher in RA than OA in serum (*p* < 0.001) and synovial tissue (*p* < 0.001) MMP14 and VEGF were higher in RA than OA in FLS (*p* < 0.001) miR-150-5p expression was lower in RA than OA in serum, synovial tissue, and FLS (*p* < 0.001) Exo150 downregulated MMP14 and VEGF expression in RA FLS and inhibited migration and angiogenesis *in vitro* (*p* < 0.001)
Wang L. (2018) [31]	25 RA** 25 HC	No therapy	Treg frequency was decreased in RA vs. HC and inhibited by RA-exosomes RA exosomes resulted in a decreased Treg ratio vs. HC exosomes miR-17, miR-19b, and miR-121 were overexpressed in RA miR-17 had a negative correlation with Treg miR-17 inhibited expression of TGFBRII and Treg induction
Van Eijk I.C. (2009) [32]	24 RA* (9 re-evaluated after 8 weeks) 15 HC	No therapy at baseline (NSAIDs admitted) Treated for 8 weeks with SSZ, MTX and GC: 9	Total MPs were similar in RA and HC MPs exposing C1q, CRP, and SAP were higher in RA vs. HC (*p* < 0.001) At baseline, ESR and CRP correlated with MPs exposing C1q, CRP, and SAP (*p* = 0.02; *p* < 0.001; *p* = 0.003; *p* = 0.02; *p* = 0.001; *p* = 0.02) After treatment, ESR, DAS28, and CRP decreased, whereas total circulating MPs and MPs exposing complement components or activator molecules were unaffected
Cloutier N. (2012) [33]	23 RA 18 PsA	–	MPs in RA were heterogeneous in size (mostly 100–300 and 700–3000 nm) Annexin V MPs in RA were higher than PsA (*p* = 0.0004) In RA there were more MP-ICs and CD41+ MP-ICs than PsA (*p* < 0.0001; *p* = 0.0006) Blockade of CD32a did not impede mpIC formation Platelet MPs contained citrullinated epitopes and were recognized by ACPA (vimentin and fibrinogen) MPs and MP-ICs stimulated leukotriene production by neutrophils
Knijff-Dutmer E.A.J. (2002) [34]	19 RA* 9 active 10 inactive 10 HC	No anticoagulants and/or GC allowed; MTX: 6; SSZ: 5; gold: 2; HCQ: 4; LFN: 1; NSAIDs	Platelet count was normal in all 3 groups PMPs were higher in RA than in HC (*p* = 0.05), with no difference between active and inactive disease PMPs correlated with DAS28 in active RA patients (*p* = 0.05), but not with CRP or ESR
Xu D. (2018) [35]	76 RA 20 HC	–	20 miRNAs were aberrantly expressed in serum exosomes from 3 RA (2 statistically significant: miR-548a-3p and miR-6891-3p) miR-6089 was decreased in serum of RA vs. HC (*p* < 0.001) miR-6089 was reduced in PBMCs in RA vs. HC (*p* < 0.001) miR-6089 was negatively correlated with CRP, RF, and ESR (*p* < 0.001)
Marton N. (2017) [36]	20 RA** 15 PsA 19 HC	DMARDs: 96%; bDMARDs: 35%	MVs from RA and PsA could not impair osteoclastogenesis Presence of exosomes inhibited the ability of CD14+ monocytes to differentiate into TRAP+ multinucleated cells in RA and HC (*p* < 0.01), PsA-derived exosomes enhanced osteoclastogenesis (*p* < 0.05) RA and HC derived exosomes expressed higher levels of RANK than PsA (*p* < 0.05) In RA, exosomes were mostly platelet-derived (CD42b+), while MVs were more B cell (CD19+) and T cells (CD3+)
Gitz E. (2014) [37]	10 RA* 10 HC	–	CD41+ MPs were higher in RA than HC (*p* < 0.01). CLEC-2 on CD41+ was similar. GPVI on CD41+ was lower in RA than HC (*p* < 0.01, *p* < 0.01). Soluble GPVI was higher in RA than HC (*p* < 0.01)
Greisen S.R. (2017) [38]	5 RA* 5 HC	Treatment according to ACR 2015 guidelines	EVs were present in plasma and SF RA: they could be isolated from PBMC and SFMC. EV size distribution did not differ between RA and HC cell cultures. PD-1 is present in RA patients both in soluble form and in association with EVs RA vs. HC PBMC: 12 miRNA, linked to PD-1/PD-ligands, were found different (*p* < 0.05) miRNA content in EVs from RA SFMC, RA, and HC PBMC was different: a minor number of PD-1, PD-L1, and PD-L2 related miRNA changed in EVs generated from stimulation of RA SFMC Data suggested that EVs transfer the co-inhibitory receptor PD-1 to cells in the microenvironment Lymphocytes co-cultured with EVs had an increased PD-1 expression (*p* < 0.05) The number of lymphocytes co-cultured with EVs from RA PBMCs was higher than those co-cultured with HC PBMCs (*p* < 0.05)
Gyorgy B. (2012) [39]	Plasma: 12 RA** 9 OA SF: 8 RA** 8 OA 10 oligoarticular JIA	–	Data on 3 patients per group. In SF pellets, there were other particles besides MVs (proteins, immunecomplexes). Besides canonical MV proteins, many plasma proteins (albumin, transferrin, fibrinogen, prothrombin, haptoglobin) and immunocomplex related proteins (complement, immunoglobins) were present. No difference in the 3 groups. Data on 8 patients for RA group. Annexin A MVs were not signifincatly elevated in RA vs. OA. Most MVs derived from B and T cells in RA e OA SF, lower monocyte and platelet MVs were present. CD3+ MVs were higher in RA than OA SF (*p* = 0.027). CD8+ MVs were higher in RA than OA SF (*p* = 0.009). B cell–derived MVs were lower in JIA than OA and RA SF (*p* = 0.009, *p* = 0.004). CD3 and CD8 MVs were undetectable in RA and OA plasma (*p* < 0.001), indicating local production. RANK and RANK-L associated MVs were found in all 3 groups. T and B cell-derived MVs correlated to RF (*p* = 0.002, *p* = 0.001). T and B cell–derived MV count correlated (*p* < 0.001). CD41 EVs correlated with disease duration (*p* = 0.008). SF cell number showed weak association with CD3 and CD8 MV counts (*p* = 0.039, *p* = 0.017)
Fan W. (2017) [40]	34 RA** 33 OA 42 HC	–	CD4 MPs were higher in RA than OA and HC (*p* < 0.0052, *p* < 0.0007). In CD4 MPs: CD161/CD39 MPs were higher in RA than OA and HC (*p* < 0.0045, *p* < 0.0013), CD73/CD39 MPs were higher in RA than OA and HC (*p* < 0.0312, *p* < 0.0065) CD161/CD39 MPs were positively correlated with DAS28, SJC and RF (*p* = 0.007, *p* = 0.003, *p* = 0.011). CD73/CD39 MPs were negatively correlated with DAS28, SJC, and RF (*p* = 0.004, *p* = 0.018, *p* = 0.014) In RA FLSs culture, CD161/CD39 MPs increased CCL20 production (*p* < 0.002), CD73/CD39 MPs increased CCL17 and CCL22 production (*p* < 0.0018, *p* < 0.0022). No effects for HC MPs In RA PBMCs culture CD161/CD39 MPs increased IL-17 production (*p* < 0.0045), CD39/CD73 MPs inhibited IL-17 production and increased IL-10 production (*p* < 0.0217, *p* < 0.0156). No effects for HC MPs
Umekita K. (2009) [41]	20 RA* (6 received LCAP) 10 HC	PDN: 12 (mean dosage 6.4 mg/die); MTX: 8; SSZ: 5; bucillamine: 4; tacrolimus: 3; LFN: 1	Mean CD61 and CD42a were higher in RA than HC (*p* < 0.0001, *p* < 0.0001). No difference for CD66b and CD16 MPs in RA and HC CD61 MPs correlated with CRP, ESR, DAS28 (*p* = 0.02, *p* = 0.002, *p* = 0.0126). CD42a correlated with ESR (*p* = 0.01) After the first section of LCAP: CD61 and CD42a MPs decreased (*p* < 0.05), CD66b and CD16 MPs increased (*p* < 0.01) After 8 weeks of follow-up (5 LCAP): DAS28 and DAS28-CRP decreased significantly, mean numbers of CD61 and CD42a MPs decreased (*p* = 0.004, *p* = 0.005), mean numbers of CD66b and CD16 MPs did not change.
Messer L. (2009) [42]	7 RA* 5 OA 3 microcrystalline arthritis 5 ReA	PDN: 100%; MTX: 100%; IFX: 14%	MPs from SF were higher in RA and microcrystalline arthritis than OA and ReA (*p* < 0.05, *p* < 0.05) The ability of RA FLS to induce BAFF, IL-6, and IL-8 after stimulation of MPs is independent from MP origin (OA or RA) RA FLS released TSLP protein and SLPI after MPs exposure (not only derived from RA SF)
Jüngel A. (2007) [43]	9 RA* 7 OA	–	RA and OA synovial fibroblasts, incubated with MPs, produced PGE2 dose-dependently (*p* < 0.005). PGE_2_ was not present in MPs. No difference according to MPs origin Incubation of RA and OA synovial fibroblasts with MPs did not increase phospholipase A2 release. MPs dose-dependently induced COX-2 and mPGES-1 mRNA in RA and OA synovial fibroblasts (*p* < 0.05, *p* < 0.05), not COX-1, mPGES-2, and cytosolic PGES. No difference according to MPs origin Upregulation of PGE_2_ was mainly mediated via COX-2 (*p* < 0.05) MPs activated NF-kB and AP-1 signaling in synovial fibroblasts. There was a significant reduction of the induction of mPGES-1 by MPs in RA synovial fibroblasts, when NF-kB and AP-1 were inhibited (*p* < 0.05, *p* < 0.05) MPs increased p38 and JNK, but only the inhibition of JNK caused a significant reduction in PGE_2_ production MPs transferred arachidonic acid into sinovial fibroblasts
Wang Y. (2017) [44]	76 RA 20 HC	–	RA miR-548-3p was downregulated in serum and PBMCs exosomes vs. HC (*p* < 0.001) Low levels of miR-548a-3p were associated with higher levels of CRP, RF, and ESR (*p* < 0.001) miR-548a-3p was involved in TLRs-mediated response (in particular TLR4 and NF-kB)
Szabó-Taylor K.É. (2017) [45]	71 RA** 54 HC	–	Exofacial thiol EV levels decreased upon LPS stimulation of U397 cells (*p* < 0.05). Monocytes from RA (*n* = 6) released EVs with lower exofacial thiol content vs. HC (*p* < 0.001) Plasma-derived EV esofacial thiols did not show any difference between RA and HC (CD9, CD41a, annexin V), while total plasma thiol levels were lower in RA vs. HC (*p* < 0.0001) Higher number of plasma exofacial peroxiredoxin-1 positive EVs in RA (*n* = 16) vs. HC (*p* < 0.05)
Headland S.E. (2015) [46]	7 RA (blood+SF) 22 RA (SF) HC	No treatment: 2; GC: 3; DMARDs: 3; bDMARDs: 3 No treatment: 0; GC: 3; DMARDs: 13; bDMARDs: 0	There were more total, CD66b, CD14, and CD3 MVs in SF than in plasma (*p* = 0.005, *p* = 0.016, *p* = 0.022, *p* = 0.008). SF MVs had more annexin A1 than plasma MVs, with more annexin A1 MV of neutrophil origin. In SF, there were more neutrophil MVs than monocyte or T cell MVs (*p* < 0.001, *p* = 0.001), with more annexin A1 (*p* < 0.001 for both)
Chen X.M. (2020) [47]	15 RA** 30 PsA 15 psoriasis 15 gout 15 HC	No therapy in the previous 4 weeks	198 and 31 microRNAs were up- and downregulated, respectively, in RA vs. HC 36 commonly expressed microRNAs were identified (29 up- and 7 downregulated) vs. HC 5 microRNAs (hsa-miR-151a-3p, hsa-miR-199a-5p, hsa-miR-370-3p, hsa-miR-589-5p, and hsa-miR-769-5p) were considered to be connected with the common pathogenesis of PsA, psoriasis, RA, and gout
Oba R. (2019) [48]	20 RA 20 OA 13 EBV 10 atopic dermatitis 20 HC	–	CD3 and CD4 were included in both Th1 and Th2 derived EVs Alpha and beta chains of HLA-DR were dominant in Th1 derived EV vs. Th2 derived EVs CD3+ HLA-DR+ EVs were higher in Th1 than Th2 (no diffeernce for CD3/CD4+ and CD3/CD63+) CD3+HLA-DR+ EVs were similar in RA and OA vs. HC CD3+CD4+ EVs were higher in all four diseases vs. HC (RA vs. HC: *p* < 0.05) CD3+CD8+ EVs were higher in EBV infection and lower in RA (*p* < 0.01)
Villar-Vesga J. (2019) [49]	18 RA** All positive for ACPA and/or RF 41 HC	No biologics	Platelets were a frequent source of MPs (50% in RA and 40% in HC) with platelet activation markers (CD62P, CD154, annexin V, and DIOC6). RA had more citrullinated peptides and IgG MPs than HC (*p* ≤ 0.05; *p* ≤ 0.01) Platelets from HC produced MPs when stimulated with collagen type IV, similarly to RA
Reich N. (2011) [50]	12 RA* 9 for RA synovial fibroblasts 3 for SF	MTX: 8; GC: 7; ADA: 4; ETN: 1; RTX: 2; LFN 1; IFX: 1	Expression of chemokines in RA synovial fibroblasts co-incubated with MPs from Jurkat T cells, U937 monocytes, and SF was increased (CXCL1, CXCL2, CXCL3x, CXCL5, and CXCL6) Supernatants from RA synovial fibroblasts co-incubated with MPs induced migration of ECs in transwell chamber assays vs. supernatants without MPs (*p* = 0.01) and neutralizing antibodies reduced the stimulatory effect Supernatants did not affect proliferation or viability of ECs (number of apoptotic or necrotic cells unchanged)
Michael B.N.R. (2018) [51]	23 RA** 17 OA 22 HC	No DMARDs	SF MPs were higher in RA than OA (*p* < 0.0001), SF PMPs were higher in RA than OA (*p* = 0.0472), SF non-PMPs were also higher (*p* < 0.0001) Plasma MPs were higher in RA (*p* < 0.0001) and OA (*p* < 0.01) vs. HC, plasma PMPs were higher in RA (*p* < 0.0001) and OA (*p* < 0.01) vs. HC, plasma non-PMPs were higher in RA (*p* < 0.0001) and OA (*p* < 0.01) vs. HC
Liao T. L. (2018) [52]	40 RA** with active disease (DAS28 > 3.2) after csDMARDs 20 with cronic HCV 20 without HCV	csDMARDs, ADA, ETN, GOL, RTX	miR-155 was increased in PBMCs in RA patients with HCV vs. patients without HCV (*p* < 0.001) and it suppressed HCV replication (*p* < 0.01) RA patients with HCV had higher exo-miR-155 levels vs. HCV negative (*p* < 0.01), RA patients with HCV treated with RTX had decreased exo-miR-155 expression vs. TNFi or csDMARDs (*p* < 0.05)
Rodríguez-Carrio J. (2015) [53]	13 RA 33 HC	TNFi naive; GOL: 11 or ETN: for 3 months; all on MTX; GC: 10	Tang and EPC increased after treatment, Tang reached levels similar to HC (*p* = 0.522) DAS28 decreased (*p* < 0.001) and paralleled Tang increased (*p* = 0.011) Tang increase was greater in good responders (*p* = 0.03), only good responders displayed parallel increase of Tang and EPCs (*p* = 0.037); treatment was associated with decreasing VEGF (*p* = 0.002), leptin (*p* = 0.014), SDF1a (*p* = 0.002) in the whole group, IL-8 and TNFα in good responders (*p* = 0.045) Tang-MP shedding was decreased after treatment (*p* = 0.021), especially in good responders (*p* = 0.006)
Sellam J. (2009) [54]	24 RA* 43 pSS 20 SLE 44 HC	GC less than 10 mg; MTX: 16; anti-TNF: 5; LFN: 2	Patients with pSS (*p* < 0.0001), SLE (*p* = 0.0004) and RA (*p* = 0.004) showed increased plasma levels of total MPs vs HC. No difference between pSS, RA and SLE All showed increased levels of platelet MPs (*p* < 0.0001), pSS also increased leukocyte MPs (<0.0001) and higher vs. RA (*p* = 0.015) and SLE (*p* = 0.003) Leukocytes MPs and DAS28 showed negative correlation in RA (*p* = 0.005) Total and platelet MPs were inversely correlated with sPLA2 activity in all groups (*p* = 0.0007 and *p* = 0.002)
Berckmans R. J. (2005) [55]	8 RA* 3 UA	DMARDs 4.5 in RA	MPs numbers in UA and RA were similar SF from RA and UA contained MPs of monocytic (CD14) and granulocytic (CD66e) origin and low levels of MPs from platelets and erythrocytes; MPs from B cells were present in 2 RA patients; MPs from CD8+ T cells SF MPs + FLS: increase in MCP-1 (*p* = 0.01), sICAM-1 (*p* = 0.01), IL-8 (*p* = 0.008), IL-6 (*p* = 0.042), VEGF (*p* = 0.001), RANTES (*p* = 0.031), and decrease in GM-CSF (*p* = 0.002). Total number and granulocyte-derived MPs of SF MPs and plasma MPs correlated with IL-8 (*p* < 0.0001) and MCP-1 (*p* < 0.0001); monocytes-derived MPs did not
Tsuno H. (2018) [56]	12 active RA* (DAS28 > 2.7) 11 inactive RA* (DAS28 < 2.3) 10 OA 10 HC	Active: DMARDs: 75%; MTX: 50%; bDMARDs: 8%; PDN: 41.7%) Inactive: DMARDs: 90.9%; MTX: 81.8%; bDMARDs: 18.1%	204 protein spots were detected on the gel In RA 28/204 protein spots had different intensity (*p* < 0.05), in particular 7 of these In active RA 24 spots showed ≥ 1.3-fold intensity differences vs. HC, in inactive RA 5 spots (only 2 overlapped with active RA) Six protein spots were identified, among which TLR3 showed 6-fold higher intensity in active RA group vs. the others The band intensity of TLR3 fragments (17-18 kDa) was higher in RA vs. HC
Viñuela-Berni V. (2015) [57]	55 RA* 6 remission 6 LDA 22 MDA 21 HDA 14 SLE 20 HC	DMARDS (MTX, SSZ), PDN: 31; no treatment: 20 For the 8 patients prospectically followed: MTX, SSZ, and PDN	Levels of Annexin V+ MPs derived from monocytes (CD14+), platelets (CD41a+), EC (CD62E+) and B cells (CD19+) were enhanced in HDA RA vs. HC (*p* < 0.001); no difference for LDA and HC MDA RA had enhanced levels of CD14+ and CD62E+ MPs (*p* < 0.05) Differences in urine levels of CD14+ and CD19+ between LDA and HDA Correlation between plasma and urine levels of MPs and DAS28 (*p* < 0.05) No difference in MPs levels between treated and untreated patients Decrease in plasma levels of all MPs after 4 weeks of therapy (*p* < 0.05) and urine MPs levels of CD14+, CD41+ and CD3+ MPs (*p* < 0.05) Mononuclear cells from HDA stimulated with MPs induced release of IL-1, IL-17, and TNFα, and an association between release of IL1 and TNFα and DAS28 was observed (*p* < 0.05)
Yoo J. (2017) [58]	60 RA** 30 CR (DAS28ESR ≤ 2.6) 30 non-CR (DAS28 > 2.6)	MTX for at least 6 months for all patients; GC: 4 in CR, 29 in non-CR	Six candidate proteins identified Serum and exosomal AA protein levels were higher in non-CR vs. CR (*p* = 0.001), serum and exosomal AA levels correlated (*p* = 0.001) Serum CRP correlated with serum AA in CR (*p* = 0.001) and in non-CR (*p* < 0.001) Serum CRP correlated with exosomal AA in non-CR (*p* < 0.001), but not in CR Exosomal levels of LYVE-1 were lower in non-CR vs. CR (*p* = 0.01), no difference between serum LYVE-1 levels in CR and non-CR; there was a weak correlation between serum and exosomal LYVE-1 There was a positive correlation between serum and exosomal LYVE-1 and CRP in non-CR (*p* = 0.04, *p* = 0.002) and a negative correlation between anti-CCP titer and exosomal LYVE-1 in non-CR (*p* = 0.014)
Zhang H. G. (2006) [59]	10 RA* 10 OA	–	Membrane-bound TNFα detected on exosomes was produced by RA synovial fibroblasts but not OA synovial fibroblasts; exosomes expressed only the membrane-bound TNFα but not the soluble form RA synovial fibroblasts exosomal TNFα induced cytotoxicity of L929 cells, not OA synovial fibroblasts; preincubation with a TNF antagonist blocked the effect RA synovial fibroblasts exosomes induced activation of NFkB signaling pathway in RA synovial fibroblasts, not OA synovial fibroblasts exosomes, but RA synovial fibroblasts exosomes induced NFkB in OA synovial fibroblasts; the TNF antagonist neutralized the effect RA synovial fibroblasts exosomes induced MMP-1 in RA synovial fibroblasts, less with TNFi (*p* < 0.0018), not OA synovial fibroblasts exosomes TNFα stimulated production of RA synovial fibroblasts exosomes (*p* < 0.0011), slightly for OA synovial fibroblasts exosomes; TNFi reduced the effect Coculture of RA synovial fibroblasts exosomes with CD4+T cells resulted in sustained cell proliferation and induction of IFNγ and IL-2 (not OA synovial fibroblasts exosomes) and TNFi partially reversed the effect In T cells, phosphorylated Akt was induced and NFkB activity increased

*1987 ACR criteria; **2010 EULAR/ACR criteria

*AA*: amyloid A; *ACR*: American college of rheumatology; *ADA*: adalimumab; *Anti-CCP*: anti–citrullinated protein antibodies; *ACPA*: anti-citrullinated protein antibodies; *CDAI*: clinical disease activity index; *CP*: citrullinated peptides; *CR*: clinical remission; *CRP*: C-reactive protein; *CV*: cardiovascular; *DAS28*: disease activity score on 28 joints; *DMARDs*: disease modifying anti-rheumatic drugs (b-: biological; cs-: conventional synthetic); *EBV*: Epstein-Barr virus; *EC*: endothelial cell; *EPC*: endothelial progenitor cell; *ESR*: erythrocyte sedimentation rate; *ETN*: etanercept; *EVs*: extracellular vesicles; *FLS*: fibroblast-like synoviocytes; *GC*: glucocorticoid; *GOL*: golimumab; *HAQ*: health assessment questionnaire; *HC*: healthy controls; *HCQ*: hydroxychloroquine; *HCV*: hepatitis C virus; *HDA*: high disease activity; *HMGB1*: high mobility group box 1; *HMVEC*: human microvascular endothelial cells (-D: dermal; -L: lung); *HUVEC*: human umbilical vein endothelial cells; *IC*: immunocomplexes; *IFX*: infliximab; *JIA*: juvenile idiopathic arthritis; *LCAP*: leukocytapheresis; *LDA*: low disease activity; *LFN*: leflunomide; *MDA*: moderate disease activity; *MDM*: monocyte-derived macrophages; *MPs*: microparticles; *MTX*: methotrexate; *MVs*: microvesicles; *NSAIDs*: non-steroidal anti-inflammatory drugs; *OA*: osteoarthritis; *PBMC*: peripheral blood mononuclear cells; *PDN*: prednisone; *PMPs*: platelet microparticles ; *PLA2s*: phospholipases *A2*; PsA: psoriatic arthritis; *pSS*: primary Sjögren syndrome; *RA*: rheumatoid arthritis; *ReA*: reactive arthritis; *RF*: rheumatoid factor; *RTX*: rituximab; *SAP*: serum amyloid protein; *SC*: subcutaneous; *SF*: synovial fluid; *SFMC*: synovial fluid mononuclear cells; *SJC*: swollen joint count; *SLE*: systemic lupus erythematous; *SLPI*: secretory leucocyte protease inhibitor; *SSZ*: sulfasalazine; *Tang*: angiogenic T cells; *TCZ*: tocilizumab; *TF*: tissue factor; *TJC*: tender joint count; *TNFi*: tumor necrosis factor inhibitor; *TSLP*: thymic stroma lymphopoietin; *VAS*: visual analog scale; *UA*: undifferentiated arthritis

Regarding the methods used for EV analysis, the vast majority of the studies used flow cytometry specifying the different cluster of domain (CD); more information about the methods can be found in supplementary materials (table S2).

### EV concentration

Total plasmatic EV number was higher in RA than healthy controls (HC), as reported in 4 studies for a total of more than 180 patients [19, 23, 51, 54]. However, in 3 studies for a total of 74 RA patients, the EV concentration was found similar between RA and HC [20, 24, 32]. Due to differences in techniques used for EV concentration, a meta-analysis could not be performed. Moreover, the EV number did not seem a specific biomarker as it was found similar in patients with reactive arthritis (ReA) [21], undifferentiated arthritis (UA) [55], systemic lupus erythematosus (SLE) [54], primary Sjögren syndrome (pSS) [54], and osteoarthritis (OA) [21, 22].

According to one study on 41 RA patients, total plasmatic EV concentration was not different between seronegative and seropositive RA [20]. Surprisingly, in one study on 60 RA patients, EV count was found statistically different between HC and only a subpopulation of RA seropositive patients (not for those patients positive for rheumatoid factor—RF, and anti-citrullinated peptides antibodies—ACPA, at high titer) [26].

Total EVs were higher in RA synovial fluid (SF) than HC plasma [24], OA SF [42, 51], and ReA SF [42]. RA SF EVs were at the same level when compared with RA plasma [24]. According to a different study, total EVs were higher in RA SF than in RA plasma [46].

### EV size

EVs from RA patients were heterogeneous in size, mostly 100–300 nm and 700–3000 nm [33]. According to a different study, smaller EVs (100 ± 50 nm) mostly derived from platelets (CD42+), while larger EVs (100–1000 nm) were more of B (CD19+) and T cells (CD3+) origin [36].

Plasmatic EV size was not different in RA as compared with HC [20, 38]. Moreover, plasmatic EV size was found similar between seronegative and seropositive RA [20]. Another study found that RA seropositive for RF and ACPA (but not if seropositivity was at high titer) had a decreased proportion of 0.1–1 μm and an elevated proportion of 1–3 μm and 3–6 μm EVs, when compared with HC [26]. Moreover, EVs from seropositive individuals had higher frequencies and wider distribution of IgM+ and IgG+ EVs [26].

Of note, some of the aforementioned studies on EV dimensions could arise concerns about the methodology used (e.g., aggregation of EVs could not have been considered). Moreover, methods reporting EVs size are often not compliant with the latest recommendations [10] and this makes comparison across different studies insidious.

### EV cell origin

Many of the studies included in this systematic review focused on surface molecules with the intent of unraveling EV origin and function. Therefore, various aspects were studied: platelets (CD41, CD42a, CD62P), leukocytes (CD45), T and B lymphocytes (CD3, CD4, CD8, CD20, CD154), granulocytes and monocytes (CD14, CD16, CD66b), endothelial cells (CD146, CD62P), and cell adhesion markers (CD31, CD61).

CD3+/HLA-DR+ [48], CD3+/CD4+ [48], CD146+ [19], CD66b+ [19], CD31+ [19, 23], CD41+ [37], CD42a [41], CD61+ [34, 41, 51, 54], and CD45+ [29] plasmatic EVs were found increased in RA when compared with HC. CD41+ [23], CD45+ [23, 54], CD66b+ [41], CD62P+ [49], CD154 [49], and CD16+ [41] plasmatic EVs were found similar between RA and HC. Of note, in a study on 55 RA patients, CD14+, CD41+, CD62E+, and CD20+ EVs were higher only in patients with high disease activity as compared with HC [57].

On the contrary, according to a different study, CD20+, CD4+, CD8+, CD14+, CD66b+ plasmatic EVs were not detectable in RA, as well as in OA patients [29]. CD45+ and CD61+ EVs were found at higher levels in RA than OA [29], whereas no difference was found for CD3+/HLA-DR+, CD3+/CD4+, CD3+/CD8+ EVs between RA and OA [48]. Moreover, RA patients showed lower levels of CD45+ EVs than pSS [54] and of CD3+/CD8+ plasmatic EVs, when compared with EBV infection [48].

In RA SF, CD4+ [55], CD41+ [25], CD66+ [28, 55] and CD14+ [28, 55] EVs were found abundant, whereas glycophorin A [28, 55], CD4+ [28], CD61+ [28, 55], CD8+ [28, 55], and CD20+ [28, 55] EVs were low. These differences were not specific for RA, since they were also found in non-RA arthritis SF [28].

RA SF contained more CD61+ [51], CD45+ [29], CD3+ [39], CD4+ [29, 40], CD4+/CD161+/CD39+ [40], CD4+/CD73+/CD39+ [40], CD8+ [29, 39], CD14+ [29], and CD66+ [29] EVs than OA SF, whereas there was no difference for CD20+ EVs [29]. Annexin A1+, CD66b+, CD14+, and CD3+ EVs were higher in RA SF than RA plasma [46]. SF CD66b+ EVs were more abundant than CD14+ and CD3+ EVs [46], whereas CD3+ and CD8+ EVs were absent in plasma [39].

### EV content

More citrullinated peptides and IgG were found in EVs, when compared with HC, in a study on 18 RA patients [49]. Citrullinated and not-citrullinated proteins were also present in RA, OA, and ReA, but fibronectin/IgG immunocomplexes (ICs) were found only in RA EVs [21]. According to another study, platelet EVs contained citrullinated epitopes, which were recognized by ACPA (vimentin and fibrinogen) [33]. Plasmatic EV protein content was not different in seronegative and seropositive RA [20]. IgM-RF was found in EVs in about half of RA patients seropositive for RF [20]. Burbano C. et al. found differences according to seropositivity: IC-EVs were higher in seropositive patients, whereas there was no difference in seronegative patients, as compared with HC [26]. Moreover, a similar difference between seronegative and seropositive patients was observed concerning systemic inflammation and EVs positive for IgG, IgM, CD41, and citrulline [26]. Furthermore, there were more IC-EVs in RA patients than in psoriatic arthritis (PsA) [33]. EVs with IgM and IgG were higher in RA SF than RA and HC plasma [24].

EVs with C1q, C3, and C4 were higher in RA SF than HC and RA plasma, while no differences were found between RA and HC plasma [24]. On the contrary, plasmatic EVs with C1q were higher when compared with HC in a study on 24 RA patients [32].

EVs with C-reactive protein (CRP) and serum amyloid P (SAP) were not different between RA and HC [24], whereas EVs with CRP and SAP were higher, as compared with HC in another study [32]. Serum and EV amyloid A (AA) levels were higher in patients with active disease than in patients in clinical remission [58]. Moreover, serum and EVs AA levels correlated with each other [58].

We found 6 studies that reported results on EVs miRNA. miR-150-5p expression was lower in RA than OA [30]. miR-6089, miR-6891-3p, and miR-548-3p were decreased in the serum of RA as compared with HC [35, 44]. miR-17, miR-19b, and miR-121 were overexpressed in RA [31]. Moreover, miR-6089 was found to negatively correlate with CRP, RF, and ESR [35]. Fan W. et al. found that 36 miRNAs (see Table 1 for details) were differently regulated in RA compared with HC [47]. In this study, 5 miRNAs (hsa-miR-151a-3p, hsa-miR-199a-5p, hsa-miR-370-3p, hsa-miR-589-5p, and hsa-miR-769-5p) were present in different forms of inflammatory arthritis (PsA, RA and gout) [47]. Moreover, 12 miRNAs, linked to programmed death (PD)-1/PD-ligands, were identified [38].

Thrombin-generating capacity (factor VIIa) was higher for SF EVs than plasma from RA patients and HC. No tissue factor (TF) antigen was present on SF EVs despite they were able to initiate TF-mediated thrombin generation [28]. C-type lectin-like receptor 2 (CLEC-2) on CD41+ EVs were similar, whereas GPIV on CD41+ were higher in RA than HC [37].

In RA patients, plasmatic EVs were found strongly bound to annexin V [22] and at a higher concentration than HC [29] and PsA [33]. This was not confirmed by a different study, since Annexin V+ EVs did not differ between RA and HC [49]. RA SF EVs boundless annexin V as compared with plasmatic EVs [28] and their number was not significantly elevated, as compared with OA [39]. Moreover, RA SF contained more annexin V+ [29] EVs than OA SF.

Furthermore, evidence from single studies suggested a potential role for PD-1 [38], TNF-α [59], RANK [36], TLR4 [44], and TLR3 [56].

### EV biological effect

Many studies focused on EV effects on pathways related to inflammation. Plasmatic EVs and IC-EVs enhanced adhesion molecules (ICAM-1, ICAM-2), inflammatory cytokines (IL-6, IL-8), and chemokines (CCL-2, CCL-5) from endothelial cells increasing vascular permeability [22] and leukotriene release from neutrophils [33].

Plasmatic EVs from seropositive patients [26] and plasmatic EVs from patients with high disease activity [57] stimulated mononuclear phagocytes to release pro-inflammatory cytokines: TNFα [26, 57], IL-6 [26], IL-17 [57], and IL-1 [26, 57].

RA fibroblast-like synoviocytes (FLS) induced a decrease in GM-CSF [55] and an increase in MCP-1 [55], sICAM-1 [55], VEGF [55], RANTES [55], BAFF [42], IL-6 [42, 55], and IL-8 [42, 55] after stimulation with SF EVs. In one of the two studies, this ability was independent from EV origin (OA or RA) [42].

EVs from Jurkat cells stimulated the induction of several chemokines (CXCL1, CXCL2, CXCL3x, CXCL5, and CXCL6) [50] in RA synovial fibroblasts. Moreover, EVs in RA synovial fibroblasts were demonstrated to play a role in MMP-1, IFN-γ, and IL-2 secretion [59]: as expected, these biological effects were partially reduced with the exposure of TNF inhibitors [59]. RA synovial fibroblasts EVs promoted NFkB signaling pathway in both RA and OA synovial fibroblasts [59]. RA synovial fibroblasts incubated with EVs from HC produced dose-dependently PGE2 regardless of EVs origin without increasing phospholipase A2 [43]. EVs dose-dependently induced COX-2 and mPGES-1 mRNA in RA synovial fibroblasts, but not COX-1, mPGES-2, and cytosolic PGES [43]. Moreover, EVs were also able to transfer arachidonic acid from leukocytes into synovial fibroblasts [43]. Levels of total and platelet plasmatic EVs were inversely correlated with secretory phospholipase A2 (sPLA2) activity in one study [54].

SF CD161+/CD39+ EVs increased CCL20 production, SF CD73+/CD39+ EVs increased CCL17 and CCL22 synthesis in RA fibroblasts, whereas SF CD161+/CD39+ EVs increased IL-17 production, and SF CD39+/CD73+ EVs reduced IL-17 and increased IL-10 production in PBMC in RA [40].

EVs were also found involved in intracellular pro-inflammatory pathways, in particular NFkB. EVs from HC activated NF-kB and AP-1 signaling in RA synovial fibroblasts and they increased p38 and JNK, but only the inhibition of JNK caused a significant reduction in PGE2 production [43].

RA IC-EVs promoted more macrophages differentiation towards a pro-inflammatory profile (M1-like) than HC [27]. Macrophages differentiated with RA IC-EVs were resistant to repolarization to M2-like after treatment with IL-4 [27]. Macrophages were also able to enhance T and B cells and prevent B cell death [27]. Plasmatic RA EVs inhibited T-reg, possibly through miRNAs (miR-17) [31].

Plasmatic CD14+ and CD41+ EVs from RA patients showed an anti-angiogenic effect, while CD62E+ and CD144+ EVs promoted endothelial activation [19]. RA plasmatic EVs increased dose-dependently apoptosis and autophagy of endothelial cells [23]. In vitro evidence suggested that SF EVs were able to induce ECs migration without affecting ECs proliferation or viability [50]. Exo150 downregulated the expression of MMP14 and VEGF in RA FLS and inhibited migration and angiogenesis in vitro [30].

One study provided interesting but non-conclusive insights about the role of exofacial thiol EVs and oxidative stress resistance that could also play a role in RA [45].

### EVs and disease characteristics

Plasmatic CD146+ EVs [19] and SF CD41+ EVs [39] levels correlated with disease duration. SF CD66b+ EVs were more abundant in established RA than early RA [29], and they were associated with the age at diagnosis [19].

A positive association was found between RF and plasmatic CD14+ [19], SF CD3+ [39], SF CD4+ [39], SF CD8+ [39], and SF CD4+/CD161+/CD39+ EVs [40]. Conversely, a negative association was observed for RF and SF CD73+/CD39+ EVs [40]. Seropositive RA patients were found to have more CD41+ EVs [26], whereas seronegative had more CD105+ EVs [26]. On the contrary, according to a different study, there was no difference regarding plasmatic EV profile between serological RA phenotypes [29]. Regarding EVs content, low levels of serum exosomal miR-548a-3p were associated with higher levels of RF [44].

SF CD3+, CD4+, and CD8+ EVs did not correlate with ACPA [39]. On the other side, ACPA positive patients had greater levels of SF annexin V+/CD45+ EVs. Moreover, a weak correlation was observed between ACPA titer, CD4+ EVs, and annexin V+/CD45+ EVs [29].

SF annexin V+ EVs and platelet-derived EVs were increased in RA patients with extra-articular symptoms [29]. Total plasmatic EV number was higher in RA than in patients with cardiovascular risk factors and their concentrations correlated with the number of traditional cardiovascular risk factors [19].

Plasmatic CD14+ and CD62E+ EVs and urinary CD14+ and CD19+ EVs differed between patients in low or high disease activity [57]. Moreover, an association was found between disease activity score on 28 joints (DAS28), plasma, and urine EVs [57].

According to another study, patients with active disease displayed an association between LYmphatic Vessel Endothelial hyaluronic acid receptor-1 (LYVE-1), both in serum and in EVs, and CRP, ACPA titer, and exosomal AA [58].

Many studies, except one [34], reported an interaction between plasmatic EVs or their content—such as miRNAs—and plasmatic inflammatory markers (ESR and CRP) [19, 20, 32, 41, 44, 58]. Four papers included tender and swollen joint count in their analysis with different outcomes: plasmatic EVs [19, 23] or SF EVs [40] influenced the joint count. Conversely, in a different study, there was no statistical correlation between plasmatic EVs and joint count [20].

One study highlighted a significant association between plasmatic EVs containing TNFα and disease activity (DAS28 or clinical disease activity index—CDAI) [23]. Several studies detected a positive correlation between DAS28 and plasmatic [19, 20, 34, 41, 57], SF [40] or urinary [57] EVs. This association was not confirmed in other four studies concerning plasmatic EV [41, 54] and SF EVs [39, 40]. Platelet EVs were similar in active and non-active RA patients [34].

### Effects of therapy on EVs

Patients treated with tocilizumab displayed lower levels of plasmatic CD3+CD31+ and CD66b+ EVs, whereas patients receiving methotrexate had decreased levels of plasmatic CD3+CD31+ EVs [19]. Adversely, another study did not find any difference in plasmatic and urinary EVs levels between treated and untreated subjects [57].

Four studies reported a decrease in either plasmatic EVs [23, 53] or urinary EVs [57] after treatment with biological disease-modifying anti-rheumatic drugs (DMARDs) or leukocytapheresis [41], whereas one paper did not report an influence of conventional synthetic (cs)-DMARDs on total plasmatic EVs [32].

A decreased exo-miR-155 expression was found in patients HCV positive treated with rituximab as compared with subjects treated with anti-TNFα or csDMARDs [52].

Of note, in these studies, RA populations enrolled are extremely heterogenous (*e.g.* different disease activity and therapies) and, for this reason, caution is needed in drawing conclusions.

## Discussion

**Fig. 2:**
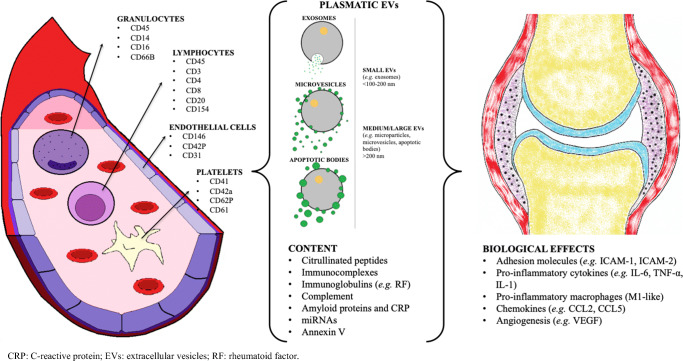
Overview of the possible role of EVs in RA. CRP: C-reactive protein; EVs: extracellular vesicles; RF: rheumatoid factor

We included and reported the results of 41 studies in this systematic review. An overview of the results and a possible role of EVs in RA is reported in Fig. 2.

The total plasmatic EV concentration seems increased in RA when compared with HC, whereas plasmatic EV size was not found dissimilar between the two groups. As already highlighted, some of the studies reporting EV size might not have considered possible shortcomings of methodology. Inconsistency among the included studies could also be explained by the relatively small population enrolled. Moreover, the total plasmatic EV number was found similar in RA and other inflammatory (i.e., SLE, UA, or ReA) and non-inflammatory conditions (i.e., OA). Conversely, RA EV concentrations in SF were described at higher levels when compared with HC, OA, and ReA.

The kaleidoscopic effects of EVs on pro-inflammatory pathways, coagulation, and angiogenesis were explored in several studies. Plasmatic EVs subpopulations, based on surface molecules and linked to numerous biological effects (e.g., cell adhesion, immune system, platelet function, vascular system, and hematopoiesis), were found higher in RA than HC, even though this was not confirmed by all studies. Interestingly, in one study, similar results were found only for high disease activity RA. Moreover, different EV subtypes were found dissimilar according to disease characteristics, such as disease duration and age at diagnosis. Plasmatic EVs and IC-EVs enhanced inflammatory pathways and cytokines (e.g., IL-6); this was true especially for EVs derived from seropositive and high disease activity patients. Studies investigating whether therapies affect EVs yielded contrasting results. Nevertheless, some in vitro and in vivo evidence could suggest a possible role of inflammatory mediators, targeted by biological therapies (e.g., TNF-α and IL-6), in the pathogenesis mediated by EVs. Despite several studies found a correlation between EVs and disease activity, this could not be confirmed in other ones. The differences observed between RA and HC were less marked when RA patients were compared with OA. Moreover, some evidence suggested that EVs containing citrullinated peptides were more abundant in RA than HC but these data were not specific for RA when compared with other arthritides.

Likewise, RA SF showed more abundant EVs derived from immune cells and platelets when compared with OA, but this could not be confirmed with respect to other non-RA inflammatory arthritides.

A limited number of studies, taking into consideration miRNAs, showed that their expression was different in RA versus HC. Furthermore, some data suggested that these differences were not specific for RA since they were not dissimilar from other pathological conditions (e.g., PsA).

EVs seem to enhance the inflammatory process, but their role is not specific for RA. As expected, since RA has the joints as the main target, studies on SF and synovial fibroblasts uncovered differences more specific to RA and, consequently, studies on them are more promising.

Even though a multitude of information could be obtained, definitive conclusions about the role of EVs in RA can difficultly be drawn. Indeed, the analyzed articles varied greatly in methodology and this makes direct comparisons challenging. Moreover, some studies were conducted on small and poorly characterized (e.g., seropositivity, therapy) populations. Various narrative reviews on this topic have been published so far, but they seem to lean excessively on the positive findings partially neglecting the negative ones.

In conclusion, EVs could contribute to better understand RA pathogenesis and they could represent a possible therapeutic target [60]. Moreover, EVs could be of help in the diagnosis (inflammatory vs. non-inflammatory joint diseases), prognosis (e.g., extra-articular involvement) and therapeutic response. Studies focusing on this topic are urged to follow rigorous methodology [10], so that the scientific community can compare them and draw translatable observations.

## Supplementary information

ESM 1 (DOCX 43.9 kb)

## Data Availability

The whole review process has been performed according to PRISMA statements.
