# Supplementary material for: The role of extracellular vesicles in rheumatoid arthritis: a systematic review
Source: Clin Rheumatol. 2021 Feb 5;40(9):3481–97. doi: 10.1007/s10067-021-05614-w (PMC8357675; doi:10.1007/s10067-021-05614-w)
Supplement: Supplementary file 1 — (DOCX 43.9 kb) [file 10067_2021_5614_MOESM1_ESM.docx]

**SUPPLEMENTARY MATERIALS**

**Table S1.** Research strategy

PUBMED

(((((((((((((((((((((rheumatoid) AND pannus)) OR (((rheumatoid) AND fibroblast) AND synovial)) OR ((rheumatoid) AND purpura)) OR ((rheumatoid) AND vasculitis)) OR ((rheumatoid) AND polyarthritis)) OR ((rheumatoid) AND fever)) OR ((rheumatoid) AND arthrosis)) OR ((rheumatoid) AND syndrome)) OR ((rheumatoid) AND inflammation)) OR ((rheumatoid) AND disease)) OR ((rheumatoid) AND arthritis)) OR ((arthritis) AND (((autoimmune) OR auto immune) OR auto-immune))) OR ((inflammatory) AND arthritis)) OR ((erosive) AND arthritis))) AND ((((((((exosom*) OR microvesicl*) OR ((apoptotic) AND ((bodies) OR body))) OR ((circulating) AND microparticl*)) OR ((((cell derived) OR cell-derived)) AND microparticl*)) OR ((((extracellular) OR extra cellular)) AND vesicl*)) OR ((membrane) AND microparticl*)) OR ((((procoagulant) OR pro-coagulant)) AND microparticl*)))) NOT review[Publication Type])) AND English[Language]

EMBASE

(rheumatoid AND pannus OR (rheumatoid AND synovial AND fibroblast) OR (rheumatoid AND purpura) OR (rheumatoid AND vasculitis) OR (rheumatoid AND polyarthritis) OR (rheumatoid AND fever) OR (rheumatoid AND arthrosis) OR (rheumatoid AND syndrome) OR (rheumatoid AND inflammation) OR (rheumatoid AND disease) OR (rheumatoid AND arthritis) OR (arthritis AND (autoimmune OR (auto AND immune) OR 'auto immune')) OR (arthritis AND inflammatory) OR (arthritis AND erosive)) AND (exosom* OR microvesicl* OR (apoptotic AND (body OR bodies)) OR (microparticl* AND circulating) OR (microparticl* AND (cell AND derived OR 'cell derived')) OR (vesicl* AND (extracellular OR (extra AND cellular))) OR (microparticl* AND membrane) OR (microparticl* AND (procoagulant OR 'pro coagulant'))) AND ('article'/it OR 'article in press'/it OR 'editorial'/it OR 'letter'/it) AND [english]/lim

SCOPUS

( ( ( TITLE-ABS-KEY ( rheumatoid ) )  AND  ( TITLE-ABS-KEY ( synovial ) )  AND  ( TITLE-ABS-KEY ( fibroblast ) ) )  OR  ( ( TITLE-ABS-KEY ( rheumatoid ) )  AND  ( TITLE-ABS-KEY ( pannus ) ) )  OR  ( ( TITLE-ABS-KEY ( rheumatoid ) )  AND  ( TITLE-ABS-KEY ( arthritis ) ) )  OR  ( ( TITLE-ABS-KEY ( rheumatoid ) )  AND  ( TITLE-ABS-KEY ( purpura ) ) )  OR  ( ( TITLE-ABS-KEY ( rheumatoid ) )  AND  ( TITLE-ABS-KEY ( vasculitis ) ) )  OR  ( ( TITLE-ABS-KEY ( rheumatoid ) )  AND  ( TITLE-ABS-KEY ( polyarthritis ) ) )  OR  ( ( TITLE-ABS-KEY ( rheumatoid ) )  AND  ( TITLE-ABS-KEY ( fever ) ) )  OR  ( ( TITLE-ABS-KEY ( rheumatoid ) )  AND  ( TITLE-ABS-KEY ( arthrosis ) ) )  OR  ( ( TITLE-ABS-KEY ( rheumatoid ) )  AND  ( TITLE-ABS-KEY ( syndrome ) ) )  OR  ( ( TITLE-ABS-KEY ( rheumatoid ) )  AND  ( TITLE-ABS-KEY ( inflammation ) ) )  OR  ( ( TITLE-ABS-KEY ( rheumatoid ) )  AND  ( TITLE-ABS-KEY ( disease ) ) )  OR  ( ( TITLE-ABS-KEY ( chronic ) )  AND  ( ( TITLE-ABS-KEY ( autoimmune ) )  OR  ( TITLE-ABS-KEY ( auto-immune ) )  OR  ( TITLE-ABS-KEY ( auto  AND  immune ) ) )  AND  ( TITLE-ABS-KEY ( arthritis ) ) )  OR  ( ( ( TITLE-ABS-KEY ( autoimmune ) )  OR  ( TITLE-ABS-KEY ( auto-immune ) )  OR  ( TITLE-ABS-KEY ( auto  AND  immune ) ) )  AND  ( TITLE-ABS-KEY ( arthritis ) ) )  OR  ( ( TITLE-ABS-KEY ( inflammatory ) )  AND  ( TITLE-ABS-KEY ( arthritis ) ) )  OR  ( ( TITLE-ABS-KEY ( erosive ) )  AND  ( TITLE-ABS-KEY ( arthritis ) ) ) )  AND  ( ( ( ( TITLE-ABS-KEY ( pro-coagulant ) )  OR  ( TITLE-ABS-KEY ( procoagulant ) ) )  AND  ( TITLE-ABS-KEY ( microparticl* ) ) )  OR  ( ( TITLE-ABS-KEY ( membrane ) )  AND  ( TITLE-ABS-KEY ( microparticl* ) ) )  OR  ( ( TITLE-ABS-KEY ( circulating ) )  AND  ( TITLE-ABS-KEY ( microparticl* ) ) )  OR  ( ( ( TITLE-ABS-KEY ( cell  AND  derived ) )  OR  ( TITLE-ABS-KEY ( cell-derived ) ) )  AND  ( TITLE-ABS-KEY ( microparticl* ) ) )  OR  ( ( ( TITLE-ABS-KEY ( extracellular ) )  OR  ( TITLE-ABS-KEY ( extra-cellular ) ) )  AND  ( TITLE-ABS-KEY ( vesicl* ) ) )  OR  ( ( TITLE-ABS-KEY ( apoptotic ) )  AND  ( ( TITLE-ABS-KEY ( body ) )  OR  ( TITLE-ABS-KEY ( bodies ) ) ) )  OR  ( TITLE-ABS-KEY ( exosom* ) )  OR  ( TITLE-ABS-KEY ( microvesicl* ) ) )  AND  ( EXCLUDE ( SUBJAREA ,  "CHEM" )  OR  EXCLUDE ( SUBJAREA ,  "CENG" )  OR  EXCLUDE ( SUBJAREA ,  "COMP" )  OR  EXCLUDE ( SUBJAREA ,  "ENGI" )  OR  EXCLUDE ( SUBJAREA ,  "MATE" )  OR  EXCLUDE ( SUBJAREA ,  "AGRI" )  OR  EXCLUDE ( SUBJAREA ,  "PHYS" ) )  AND  ( EXCLUDE ( DOCTYPE ,  "re" ) )  AND  ( LIMIT-TO ( LANGUAGE ,  "English" ) )

**Table S2.** Characteristics of the included studies.

| **References** | **Country** | **Study design** | **Setting** | **Outcomes mesured** | **Patients enrolment strategies** | **Methods for EVs** | **Age** [years] | **Gender** [F:M] |
| --- | --- | --- | --- | --- | --- | --- | --- | --- |
| Rodríguez-Carrio J. (2015) [19] | Spain | Observational cross-sectional study | University hospital | MPs, CV risk factor and endothelial function | RA consecutive patients CV risk from primary care | Plasma flow cytometry (CD41, CD146, CD14, CD66b, CD3, CD31) on 10 patients per group: angiogenesis and endothelial activation | 55.0 44.3 57.4 | 90:24 25:8 38:34 |
| Arntz O.J. (2018) [20] | The Netherlands | Observational cross-sectional study | University hospital | EVs and disease specific parameters (VAS, DAS28, ESR, CRP, TJC, SJC) | HC from blood transfusion dpt. | Plasma exclusion chromotography, Nanosight particle tracking analysis and micro-BCA | 59.7 | 31:10 |
| Skriner K.  (2006) [21] | Germany | Observational cross-sectional study | University hospital | Ammount and protein content exosomes of SF | - | Exosomes on SF Electron microscopy. 2D electrophormesis-immunoblotting and mass spectrometry | - | - |
| Atehortùa L. (2019) [22] | Colombia | Observational cross-sectional study | University hospital | MPs and endothelial activation, adhesion of monocytes to endothelium, endothelial damage, cytokines and chemokines production | - | Plama flow cytometry and fluorescence microscopy (HUVEC, HMVEC-L and -D) | 56 26 Matched | 9:0 8:1 6:0 |
| Barbati C.  (2018) [23] | Italy | Observational longitudinal study | University hospital | Surface expression of TNFα, apoptosis and autophagy at baseline and after 4 months of ETN DAS28, TJC, SJC, CDAI and HAQ | - | Flow cytometry (CD31, CD41a, CD45) + EM | 58.5 58 | 19:1 20:0 |
| Birò E. (2007) [24] | The Netherlands | Case-control study | University hospital | Activated complement components and complement activator on surface on RA MPs | - | Flow citometry on plasma and SF | 57.7 Age- and sex-mathed | 6:4 Age- and sex-mathed |
| Boilard E. (2010) [25] | USA and UK | Observational cross-sectional study | University hospital | SF platelet MPs concentration | - | Flow cytometry on SF (CD41, CD45, CD15, CD3, CD14) | - | - |
| Burbano C. (2018) [26] | Colombia | Observational cross-sectional study | University hospital | EVs count, size distribution, components, effect of EVs on mononuclear phagocytes | - | moAbs flow cytometry (CD41a, CD45, CD105, CD33, CD235a, CD3, CD19, CD14, CD16, HMGB1, C1q, HLA-DR), EV-ICs, citrullinated peptide on EVs | 55 46 | 50:10 34:6 |
| Burbano C. (2019) [27] | Colombia | Observational cross-sectional study | University hospital | Effect of MPs and MP-ICs in monocytes differentation (macrophages nad lynphocyte activation) | - | EVs size, concentration, flow cytometry (CD14, CD16, CD41a, CD45, CD105, CD33, CD235a, CD3, CD19, and HLA-DR) | 45 39 Age- and sex-mathed | 28:6 29:5 Age- and sex-mathed |
| Berckmans R.J. (2002) [28] | The Netherlands | Observational cross-sectional study | University hospital | Origin and procoagulant properties of synovial MPs | - | Blood and SF. Flow citometry (CD4, CD8, CD14, CD20, CD61, CD66b, glycophorin A). Thrombin-generation test. | 62.5 52.5 Age- and sex-matched | 7:3 6:4 Age- and sex-matched |
| Michael B.N.R. (2019) [29] | India | Observational cross-sectional study | Hospital | Profiling cell lineage-specific MPs | Consecutive patients | Blood and SF. Flow citometry (annexin V APC, CD45, CD20, CD14, CD4, CD8, CD66b, CD61) | 43.6 50.6 43.4 | 35:5 27:3 26:7 |
| Chen Z. (2018) [30] | China | Observational cross-sectional study | University hospital | Effects of mir-150-5p exosomes | Patients who underwent synoviectomy or joint replacement | Plasma e synovial tissue. ExoQuick-TC quick, transmission EM and dynamic light scattering | - | - |
| Wang L. (2018) [31] | China | Observational cross-sectional study | University hospital | miR-17-dependant expression of TGFβRII by T cells and Treg induction in patients with RA vs. HC | - | Plasma. Nanosight. miRNA expression profiles in exosomes by microarray analysis, miR-17 by qRT-PCR | 60.2 | 20:5 |
| Van Eijk I.C. (2009) [32] | The Netherlands | Cross-sectional and prospective | University hospital | Concentrations of MPs exposing C1q, CRP or SAP in patients with RA vs. HC at baseline and after 8 weeks of intensive treatment | Early RA consecutive patients | Flow citometry on plasma | 51 49 | 14:10 13:2 |
| Cloutier N. (2012) [33] | Canada, France, USA, UK and Switzerland | Observational cross-sectional study | University hospital | MPs size, origin and function | Discarded material from knee diagnostic or therapeutic arthrocentesis | High sensitivity flow citometry, EM and proteomic on SF | - | - |
| Knijff-Dutmer E.A.J. (2002) [34] | The Netherlands | Observational cross-sectional study | Out-patient clinic | Platelet-derived MPs and DAS28 | Consecutive RA patients | Flow citometry on plasma | 60.6  39.5 | 15:4 |
| Xu D. (2018) [35] | China | Observational cross-sectional study | University hospital | The role of exosomal miR-6089 | RA between 9/2015 and 1/2017 | Total exosome Isolation Kit (ultracentrifugation) and microarray analysis for exosomal miRNAs | 50.2 49.1 | 55:21 14:6 |
| Marton N. (2017) [36] | Hungary and UK | Observational cross-sectional study | University hospital | Effect of EVs on osteoclastogenesis (Osteoclast differentiation inhibition) | Out-patient department from Budapest and Glasgow | Isolation by gravity-driven filtration and differential centrifugation on plasma; resistive pulse sensing technique, EM, flow citometry (CD3, CD14, CD15, CD19, CD42b, CD235a) and western blot | 58 53.1 38.4 | 17:3 7:8 11:8 |
| Gitz E. (2014) [37] | UK and Australia | Observational cross-sectional study | University hospital | CLEC-2 and GPVI on MPs CD41+ | - | Flow citometry on plasma (CD41a, CLEC-2, GPVI, CD3, C11c, CD14, CD16, CD19) | - | - |
| Greisen S.R.  (2017) [38] | Denmark and USA | Observational cross-sectional study | University hospital | Role of programmed death 1 (PD-1) and other T cell inhibition markers and EVs | Out-patient clinic | Eco-flow purification kit. NanoSight. Flow cytometry on plasma and SF (CD63). EM and EVs co-cultered with lymphocytes. miRNA on EVs | > 18 years | - |
| Gyorgy B. (2012) [39] | Hungary, UK, Switzerland | Observational cross-sectional study | University hospital | MVs in RA as compared to OA e JIA (plasma and SF) | - | SF and plasma. Flow citometry (annexin V, CD3, CD4, CD14, RANK, RANK-L). EM, NTA and MS | 60.5 53.5 50.4 64.1 9.5 | 9:3 6:3 7:1 6:2 6:4 |
| Fan W. (2017) [40] | China | Observational cross-sectional study | University hospital | Subtypes of MPs in RA, OA and HC, and their correlation to clinical features, chemokines and cytokine | - | SF. Flow cytometry (CD3, CD4, CD161, CD39, CD73) | 53.2 55.1 51.7 | 16:18 16:17 22:20 |
| Umekita K. (2009) [41] | Japan | Cross-sectional and prospective | University hospital | LCAP (one per week for 5 weeks) | - | Flow citometry (CD61, CD42a, CD66b, CD16) and EM on plasma | 53 44.5 | 20:0 10:0 |
| Messer L. (2009) [42] | France | Observational cross-sectional study | University hospital | MPs in SF (cytokines of B-lymphocyte) | Synovial tissue from knee joint arthroscopic synovietcomy | SF. FLSs negative for CD16. Quantitative determination of MPs: prothrombin assay | 66 55.2 59.7 44.4 | 4:3 3:2 1:2 1:4 |
| Jüngel A. (2007) [43] | Germany, Switzerland, USA | Observational cross-sectional study | University hospital | MPs and synovial fibroblast pro-inflammatory phenotype | Patients underwent synoviectomy or joint replacement surgery | MPs from healthy donors. Differential centrifugation. Flow cytometry (CD3, CD14, annexin V) | - | - |
| Wang Y. (2017) [44] | China | Observational cross-sectional study | University hospital | Role of serum exosomal miR-548a-3p | - | Serum and PBMCs exosomes. Total exosome isolation kit. | 50.2 49.1 | 55:21 16:6 |
| Szabó-Taylor K.É. (2017)  [45] | Hungary and UK | Observational cross-sectional study | University hospital | Redox state and aspects of EVs | - | Flow citometry (annexin V, CD9, CD63, CD41a, CD105, CD-144, CD-146, glycophorin A, CD14) | 59.9 42 | - |
| Headland S.E. (2015) [46] | Brazil, UK and USA | Observational cross-sectional study | University hospital | Neutrophil-derived MVs | - | SF and plasma. CD14, CD3, CD66b, Annexin A1 | 6:1 12:3 | 59 57 |
| Chen X.M. (2020) [47] | China | Observational cross-sectional study | University hospital | MicroRNAs from plasma exosome | - | EM, Exo Quick, Nanosight and western blot (TSG101, CD9, CD63, and CD81) | 11:4 11:19 4:11 1:14 6:9 | 50.9 45.5 53.7 43.5 49.3 |
| Oba R. (2019) [48] | Japan | Observational cross-sectional study | Lab | T cell-derived EVs | Biobank | Serum, flow citometry (CD3, CD4, CD63, IFN-γ, IL-4, IgG2, IgG1, HLA-DR), AlphaLISA, Nanosight | - | - |
| Villar-Vesga J. (2019) [49] | Colombia | Observational cross-sectional study | University hospital | Platelet-derived MPs and ICs | - | Flow citometry (CD41a, CD62P, CD154, CD63, annexin V, DIOC6). Fluoresbrite calibration grade size range kit. EM | 40-75 | 17:1 40:1 |
| Reich N. (2011) [50] | Germany and Switzerland | Observational cross-sectional study | University hospital | Expression of pro-angiogenic ELR+ chemokines in SFs co-cultured with MPs from Jurkat T cells and U937 monocytes, migration of ECs, proliferation and viability of ECs after adding supernatants from co-cultures | - | Synovial fibroblasts and SF MPs from Jurkat T cells and U937 monocytes; flow citometry (CD3, CD14, Annexin V) and EM | 47.16 | 8:4 |
| Michael B.N.R. (2018) [51] | India | Observational cross-sectional study | Clinical Hospital | Levels of Annexin-V+ MPs and PMPs (annexin-V+CD61+) in SF and plasma | Newly diagnosed DMARD-naive patients | Flow citometry (FACSAria III, Annexin V, CD61) on SF (RA, OA) and plasma (RA, OA, HC) | 42.96  52.47  44.63 | 21: 15:2 18:4 |
| Liao T. L. (2018) [52] | Taiwan | Cross-sectional and prospective | University hospital | RTX on HCV replication and exo-miR-155 transmission levels | - | Exo Quick, Immunoblotting, flow citometry (CD63), ELISA and qRT-PCR on blood | - | - |
| Rodríguez-Carrio J. (2015) [53] | Spain | Cross-sectional and prospective (3 months) | University hospital | DAS28; EPC and Tang levels; VEGF, leptin, SDF-1a, IL-8 and TNFα levels; Tang (angiogenic T cells)-MPs | - | Flow citometry (CD3, CD31, CXCR4) on plasma | 48  49 | 12:1 22:11 |
| Sellam J. (2009) [54] | France and Switzerland | Observational cross-sectional study | University hospital | Plasma levels of total, platelet and leukocyte MPs | - | Protrombinase capture assay, flow citometry (annexin V, GPIb, CD11a, CD40, CD62p) on plasma | 55 60 35.5 41 | 21:5 42:1 19:1 37:7 |
| Berckmans R. J. (2005) [55] | The Netherlands | Observational cross-sectional study | University hospital | Concentration of MCP1, IL-8, IL-6, GM-CSF, VEGF, and ICAM-1 from FLS incubated with autologus MPs from SF; concentration of mediators in SF vs plasma | Synovial tissue from small needle knee arthroscopy | SF, plasma and syonival tissue. Flow citometry (CD4, CD8, CD14, CD20,CD61, CD66e, anti-glycophorinA) | 58 56 | 4:4 0:3 |
| Tsuno H. (2018) [56] | Japan | Observational cross-sectional study | University hospital | Serum exosomal protein profile | - | ExoQuick; TEM; two-dimensional differential gel electrophoresis (2D-DIGE), mass spectometry | 62.  63.4 62.5 60.5 | 10:2 8:3 9:1 8:2 |
| Viñuela-Berni V. (2015) [57] | Mexico | Cross-sectional, prospective (8 pts, 4 weeks) | - | Proportions of MPs, DAS28, cytokine releases *in vitro* | - | Flow citometry (CD3, CD14, CD19, CD41a, CD62e, annexin V) on plasma and urine | 38.9 38 35.7 | 51:4 14:0 20:0 |
| Yoo J. (2017) [58] | Korea | Observational cross-sectional study | University hospital | Exosomal candidate proteins related to inflammatory parameters | Random selection Jan2015-Aug2015 | ExoQuick, mass spectometry, ELISA | CR 55.3 nonCR 52.8 | 60:0 |
| Zhang H. G. (2006) [59] | USA | Observational cross-sectional study | University hospital | Synovial fibroblast properties (content and biological properties) | RA and OA patients undergoing total joint replacement | Differential centrifugation, EM, mass spectometry from synovial fibroblasts (RA and OA) | 45-73  (age matched) | 10:0 10:0 |

CDAI: clinical disease activity index; CRP: C-reactive protein; CV: cardiovascular; DAS28: disease activity score on 28 joints; EC: endothelial cell; EM: electron microscopy; EPC: endothelial progenitor cell; ESR: erythrocyte sedimentation rate; ETN: etanercept; EVs: extracellular vesicles; FLS: fibroblast-like synoviocytes; HAQ: health assessment questionnaire; HC: healthy controls; HCV: hepatitis C virus; HMVEC: human microvascular endothelial cells (-D: dermal; -L: lung); HUVEC: human umbilical vein endothelial cells; IC: immunocomplexes; JIA: juvenile idiopathic arthritis; LCAP: leukocytapheresis; MPs: microparticles; MS: mass spectometry; NTA: nanotrack analysis; OA: osteoarthritis; PBMC: peripheral blood mononuclear cells; PMPs: platelet microparticles; RTX: rituximab; SAP: serum amyloid protein; SF: synovial fluid; SJC: swollen joint count; TEM: transmission electron microscopy; TJC: tender joint count; VAS: visual analogue scale.
